# Items

**Published:** 1882-11

**Authors:** 


### Items.

---

METEOROLOGICAL DATA.—For the month of Sept., 1882. H. Hall, Observer, U. S. Signal Station, Atlanta, Ga.

| | | | |
|---|---|---|---|
| BAROMETER | —Mean | 28.897 | |
| | Highest | 30.274 | on 30th. |
| | Lowest | 29.371 | on 10th. |
| | Range | 0.903 | |
| THERMOMETER | —Mean | 70.7 | |
| | Highest | 87.2 | on 2d. |
| | Lowest | 52.2 | on 23d. |
| | Range | 35.0 | |
| | Greatest Daily Range | 21.6 | on 15th. |
| | Least Daily Range | 5.5 | on 10th. |
| Humidity—Mean | | 75.5 | |
| Dew-point—Mean | | 61.8 | |
| Rainfall—Total | | 3.51 | inches. |
| | Greatest Daily | 2.30 | on 10th. |
| | Total Number of Days | 8 | |
| Wind—Prevailing Direction | | N. W. | |
| | Maximum Velocity | N. E., 41 miles. | |

---

DR. ALBERT L. GIHON, of the United States Navy, is authority for the statement that only nine States are now without State boards of health. These are Florida, Kansas, Maine, Missouri, Nebraska, Nevada, Ohio, Pennsylvania, and Vermont. Georgia, it will be perceived, is classed with the more advanced and enterprising of her sisters. This is literally true, but practically it is an error. The act creating the board in this State remains upon the statute books unrepealed; but the General Assembly, after the second year of the board's establishment, has refused to provide the necessary appropriation for its maintenance, and consequently it has no actual, but merely a nominal existence. It has been inoperative and entirely inactive for the last seven years. Hence, Georgia, to all intents and purposes, is without a State Board of Health.

---

THE *Texas Medical and Surgical Journal*, "official organ of the Texas State Medical Association," publishes, in its October number, a paper read before the State Association and referred by the Committee on Publication. Appended to the name of the author we note not only the legitimate title M.D., but, astonishing as it may seem, the words "Occulist and Aurist." It is to be hoped that this "official organ" does not represent the ethical sentiment of the medical profession in the Lone Star State. If it does, some parts of the West, no less than some parts of the East, offer inviting fields for missionary labor.

---

MISS COBBE, a contributor to the *Modern Review*, an English literary publication, exhibits a very savage disposition in her fierce assault upon the medical profession of Great Britain, in the columns of that journal. She is not very choice in her language, or very exact in her statements, but for all that, we say, let Miss Cobbe and her venom alone. The doctor's time will come bye and bye, when her vehement denunciations shall be changed into piteous cries for help. Then let her reap the benefit, not of her violence, but of the science, the art and the charity of medicine.

---

IN the next number of the REGISTER we shall have the pleasure of publishing a paper by Dr. Nathan Bozeman, of New York, entitled "Remarks touching dilatation of the vagina and replacement of the uterus and ovaries by sponge and cotton pressure."

We regret that failure to receive the manuscript has prevented its appearance in the present issue, but we feel assured that a short delay will not diminish the cordially of the reception which will be accorded it by our readers.

---

THE *Boston Medical and Surgical Journal* says: A

metropolitan journal of wide if not select circulation contains the following choice advertisement:

Wanted, immediately, a good street-talker on medicine. One who can officiate in the office during the day. A man with a good presence and long hair preferred. Address, with reference and salary wanted. (Address, etc., ———.)

We should think this want need not be long felt.

---

THE American Public Health Association held a successful session at Indianapolis, and adjourned to meet at Detroit in 1883. The following officers were elected: President, Dr. Ezra M. Hunt, of New Jersey; First vice-President, Dr. Albert L. Gihon, U. S. N.; Second vice-President, Dr. J. E. Reeves, of Wheeling, West Virginia; Treasurer, Dr. J. B. Linsley, of Nashville, Tenn.; Secretary, Dr. Azel Ames, of Boston, Mass.

---

THE weekly circulation of the *British Medical Journal*, the official organ, and the creature, of the British Medical Association, is eleven thousand copies. This fact conveys a faint idea of the power which it wields, and feebly suggests the influence which it exerts on medical men and in medical matters.

---

SINCE the forced suspension of the National Board of Health *Bulletin*, an arrangement has been perfected with the *Sanitary Engineer* to publish the weekly returns of mortality from the principal cities of the country, which formerly appeared in the *Bulletin*.

---

WE gladly welcome the *Journal of Cutaneous and Venereal Diseases* to the hospitalities of our office. It is a new candidate for professional support, issued monthly by Messrs. William Wood & Co., and edited by Drs. Henry G. Pifford and Prince A. Morrow.

---

THE yellow sulphate of mercury—turpeth mineral—is enthusiastically recommended as an emetic in spasmodic croup. Dose, two to five grains, according to the age of the child; repeated as required. The remedy is not new, but it seems to wear well.

---

SPONGING the surface of the body with a solution of quinine in alcohol—one drachm to the pint—is now recommended for excessive sweating. It is a remedy that has long yielded us good results.

---

DR. JULIUS WISE has retired from the editorial chair of the *Mississippi Valley Medical Monthly*, and removed from Memphis to St. Louis. Dr. F. L. Sim is his successor.

---

DEPAUL recommends the administration of iodide of potassium during pregnancy, in cases of contracted pelvis, with a view of lessening the size of the child.

---

THE editorial management of the *Louisville Medical News* has been assumed by Drs. L. P. Yandell and L. S. McMurty, who succeed Drs. Holland and Cottell.

---

DR. H. J. BIGELOW, of Boston, recently received a prize of 6,000 francs, from the French Academy of Medicine.

---

THE annual report of the Surgeon-General, U. S. army, is a voluminous and interesting document.

---

THE *American Journal of Physiology*, edited by D. H. Fernandes, M.D., is upon our table.

---

ENEMATA of chloral hydrate are said to be useful in the vomiting of pregnancy.

